# Smart agriculture using digital holography and artificial intelligence

**DOI:** 10.3389/fpls.2026.1871510

**Published:** 2026-09-24

**Authors:** Tessa Chiara Basso, Sara Leoni, Denis Ullmann, Adimulya Kartiyasa, Livio Ruzzante, Sylvain Schnée, Anne-Lise Fabre, Steven Hewison, Jérôme Kasparian, Nicolas Berti, Pierre-Henri Dubuis, Jean-Pierre Wolf

**Affiliations:** 1 Université de Genève, Applied Physics, Geneva, Switzerland; 2 Agroscope Changins, Mycology Group, Research Department Plant Protection, Nyon, Switzerland; 3 Université de Genève, Institut des Sciences de l’Environnement, Geneva, Switzerland; 4 Chateau Le Puy, Saint-Cibard, France

**Keywords:** artificial intelligence, bioaerosols, crop protection, digital holography, smart agriculture, vineyards

## Abstract

In viticulture, direct protection against downy and powdery mildews relies on the preventive application of fungicides, requiring growers to anticipate infection events. Decision-making is mainly supported by forecasting models driven by weather predictions. However, these decisions are inherently uncertain, as some treatments ultimately prove unnecessary, although this information only becomes available retrospectively from observed conditions. This study explores the integration of an advanced spore detection device to better target fungicide applications in vineyards, aiming to reduce the use of phytosanitary products while maintaining high grape quality. The stand-alone device uses digital holography combined with artificial intelligence (AI) for detecting and classifying airborne spores of both downy and powdery mildew. It enables the tracking of disease dynamics as well as the assessment of environmental conditions and treatment effects on spore counts. Two case studies with real-time data access in Changins, Switzerland and Château le Puy, France, are presented and revealed promising strategies for substantial reductions in fungicide use while maintaining effective disease control.

## 1 Introduction

The reduction of pesticide use in agriculture is a critical concern within environmental policies. For instance, a recent resolution by the European Parliament highlighted that 80% of member states failed to implement effective measures for rationalising pesticide usage (European Parliament, 2019). Consequently, there is a growing drive to enforce and strengthen pesticide reduction as part of the upcoming reform of the European Union’s Common Agricultural Policy. Phytosanitary treatments also represent a significant financial burden on producers (Testa et al., 2025).

In the context of vineyards, *Vitis vinifera* cultivars are widely planted across the globe. However, these grapevines are susceptible to various fungal diseases, including downy and powdery mildew, posing significant threats to both yield and grape quality. Downy mildew, caused by the oomycete pathogen *Plasmopara viticola* (PV), thrives in mild and humid climates. In contrast, powdery mildew, due to the ascomycete *Erysiphe necator* (EN), prefers warmer and drier environments. These two pathogens exhibit obligate biotrophy, meaning they rely exclusively on living host tissues for sustenance. Their polycyclic life cycles involve rapid asexual reproduction, resulting in the abundant production of spores that can contaminate vineyards and disperse widely in the environment.

To control these diseases, preventive applications of plant protection products are essential. Winegrowers typically apply fungicides from shortly after budburst (at the 3–4 leaf stage) until the onset of veraison (the color change of grape berries). This strategic approach, usually involving 7 to 12 applications over the growing season, ensures optimal plant protection and contributes to the production of high-quality grapes. Within the agricultural community, the extensive use of phytosanitary products has sparked significant concerns. The health implications due to prolonged exposure to fungicides, coupled with the persistence of residues on treated vegetation, introduce potential risks to both agricultural workers and the broader public (Sieke et al., 2018; Baldi et al., 1999). Secondly, while these chemical agents are essential for crop protection, instances of soil and underground water pollution have been documented and directly linked to their use (Derbalah et al., 2019). Lastly, non-targeted ecosystems face disruption due to widespread fungicide use, leading to a net decline in biodiversity (Benton et al., 2002).

To address these challenges, a strategic approach involves tailoring fungicide applications based on disease dynamics. Decision support systems (DSS), coupled with forecasting models for fungal diseases, play a pivotal role in achieving this optimisation. Historical statistical models for PV (Stryzik, 1983; Magnien et al., 1991; Magarey et al., 2005) and EN (Kast, 1997; Gubler et al., 1999) were first developed in the 1980s. These models offer a simple implementation and can predict complex systems without the need for an in-depth understanding of all the underlying mechanisms. In contrast, mechanistic models demand a deeper grasp of pathogen and plant biology as well as environmental influences (i.e. weather conditions). They translate each stage of the biological development cycle into algorithms. This type of model was developed for PV (Rossi et al., 2008; Dubuis et al., 2012) and EN (Legler et al., 2014). A critical challenge lies in accurately determining the primary infection, which usually results in minimal symptoms (oil spots for PV and discreet gray colonies for EN), as the epidemic often unfolds later in the season following new infection episodes. To date, fungal disease development has been predicted using cumulative climatic parameters. However, modeling efforts often overlook fluctuations in overwintering conditions as well as treatments already applied (Shishkoff, 2019).

A promising solution to improve forecasting precision is to incorporate quantitative data on the actual pathogen spore presence. This approach has already shown promise in certain pathosystems, such as powdery mildew in grapevine (Thiessen et al., 2016) or botrytis leaf blight of onion (Carisse et al., 2009). The detection of airborne spores has gained increasing prominence due to their significant variability across both space and time (Kilin et al., 2019; Schmale and Ross, 2015). To date, quantifying spore levels on a large scale has faced two primary challenges: the labor-intensive evaluation of spore-trap bands and reliance on field sampling methods involving adhesive tapes or rotorods (Schmechel et al., 1997), followed by laboratory-based loop-mediated isothermal amplification (LAMP) (Douillet et al., 2022) or quantitative polymerase chain reaction (qPCR) (Van der Heyden et al., 2021). Accurate quantification of spore inoculum at specific locations and time points is crucial for enhancing forecasting models. However, the main limitation lies in the time-consuming nature of sample analysis, typically taking one to three days, which is an impractical delay for producers who need timely responses.

Obtaining real-time information on spore load is crucial for correctly timing a fungicide treatment, or even for deciding not to apply one if the conditions for infection are not present. This is particularly true for downy mildew, for which control products must be present on the plant’s surface during the pathogen’s infectious phase (release of infectious zoospores via surface water – 12 to 24 hours). If the pathogen is able to penetrate the plant tissues, the efficacy of the treatment is significantly reduced, and the risk of an outbreak is increased.

Various alternative strategies have been developed to minimise the latency associated with sampling and laboratory analysis. These include sophisticated bioaerosol analyzers (Hill et al., 1999; Pan et al., 1999; Kiselev et al., 2013; Crosta et al., 2013; Kaye et al., 2005; Könemann et al., 2019; Forde et al., 2019; Sauvageat et al., 2020), which detect airborne pathogens, as well as hyperspectral imaging from robots, drones, or satellites (Al-Saddik et al., 2017; Mahlein, 2016; Zarco-Tejada et al., 2003; Kuswidiyanto et al., 2023; Kerkech et al., 2020; Oerke et al., 2016). Pioneering methodological developments have been provided over two decades by the research groups at Yale and ARL (Hill et al., 1999; Pan et al., 1999, Pan et al., 2024; Ai et al., 2025). In recent years, a new class of devices known as lensless microscopes has emerged, using digital holography with significantly lower power consumption and reduced costs compared to traditional fluorescence- or scattering-based particle analyzers. Although these devices have primarily found applications in point-of-care diagnostics and histology, progress has been made specifically in the context of airborne particles (Wu and Ozcan, 2018; Berg, 2022). For instance, it was demonstrated that digital holography can provide accurate PM2.5 and PM10 (Particulate Matter) measurements, comparable to reference standards. Additionally, the use of synthetic aperture illumination can enable higher-resolution imaging (Luo et al., 2015).

Detecting and classifying spores of PV and EN provides critical information for assessing disease presence and progression. However, determining a threshold at which the risk of infection is highest is essential for providing winegrowers with actionable insights. This threshold, established through aerobiological sampling, molecular detection, and environmental modeling, defines the minimum spore concentrations required for infection under conducive conditions. Airborne spores in vineyards are predominantly Cladosporium species, though typically smaller than either PV or EN spores, with concentrations typically ranging from 1000 to 5000 spores/m^3^, hence 1000 times higher than PV or EN (Magyar et al., 2009). PV spore concentrations generally range from 2–40 sporangia/m^3^, with peaks up to 500 sporangia/m^3^, influenced by climatic conditions and vineyard management (Koledenkova et al., 2022; Douillet et al., 2022). A threshold of 2.52 viable sporangia/m^3^ was determined through multi-year spore trap studies under high humidity and temperatures of 18−25 °C. LAMP assays refined detection sensitivity to 3.3 sporangia/m^3^, correlating with a disease probability exceeding 0.5, validated against droplet digital PCR (*R*^2^ = 0.52) (Brischetto et al., 2020; Hui Wang et al., 2023; Douillet et al., 2022). The risk of infection increases with spore loads, with 1–5 sporangia/m^3^ corresponding to a 50% risk and 15 sporangia/m^3^ to 90%, detected using Hirst-type spore traps at a height of 1.5 meters. EN spore concentrations follow similar seasonal trends, ranging from 2–100 conidia/m^3^ and peaking at 4,800 conidia/m^3^ under optimal developmental conditions, strongly influenced by temperature (20−25 °C), humidity, and wind speeds above 2.3 m/s (Cortiñas-Rodríguez et al., 2020). EN infection thresholds align with PV, with 5 conidia/m^3^ sufficient to initiate infections. Furthermore, phenological degree-days (PDD) between 120–160 indicate a particularly sensitive host growth stage with a high probability of infection with EN (González-Fernández et al., 2019; Martínez-Bracero et al., 2020). There are also significant differences in spread efficiency between spores, PV being much more local than EN due to their different infection modes. PV spore dispersal is rapid but limited to 50 meters, emphasising the need for site-specific monitoring (Boso et al., 2019; Martínez-Bracero et al., 2020).

In parallel, the use of AI in agriculture has dramatically expanded in recent years (Mesías-Ruiz et al., 2023; Keykhasaber et al., 2026; Subeesh and Mehta, 2021; Javed et al., 2026; Alpsalaz et al., 2025; Özupak et al., 2025), in particular by analysing images of leaves, by different methods (local imaging, including smartphone apps, multispectral, photogrammetry, remote sensing by drone, aircraft, and satellites).

In the present paper, we introduce a novel stand-alone device, using digital holography and AI, designed for real-time, *in situ* identification and quantification of airborne downy and powdery mildew spores in vineyards. Unlike most of the above-mentioned techniques, this method provides direct measurements of the actual quantity of pathogen spores in air instead of assessing an infection risk modelled with indirect data (*e.g.*, weather). It also provides real-time risk information to the producer prior to disease development unlike observations of already infected leaves. We demonstrate initial encouraging results on fungicide reduction in crops over three seasons (2021-2023) and in two different regions (Bordeaux (F) and Changins (CH)). Additional case studies will be necessary to confirm our findings and provide statistical robustness.

## 2 Methods

### 2.1 Digital holographic device

The digital holographic device illustrated in **Figure 1** was specifically designed for airborne spore detection. It uses an air inlet with a controlled flow of 1.4 m^3^/h and a concentrator that deposits the aerosols on a sapphire wafer on a spot of 10 mm in diameter. The sapphire plate had a thickness of 150 µm and a diameter of 75 mm, polished on both sides. The refractive index and transmission of sapphire at 510 nm are 1.765 (average between extraordinary and ordinary rays) and 87% (including Fresnel losses), respectively. The air inlet includes a protective mesh that prevents ingress of insects and large debris. Particles are collected by inertial deposition onto a rotating sapphire disk; imaging is performed on deposited, dry particles on the disk surface rather than on airborne particles in transit. Consequently, rainfall droplets are not imaged, and rainy periods primarily affect the bioaerosol load and not the presence of liquid droplets on the sampling substrate. The sapphire disk can be rotated on its axis to present a fresh surface every 2–3 hours, depending on the aerosol load. Every 24 hours, the disk is automatically cleaned for the next day of operation. Highly accurate rotation allows multiple images of the same sample, for super-resolution methods like ptychography (Wu and Ozcan, 2018).

**Figure 1:**
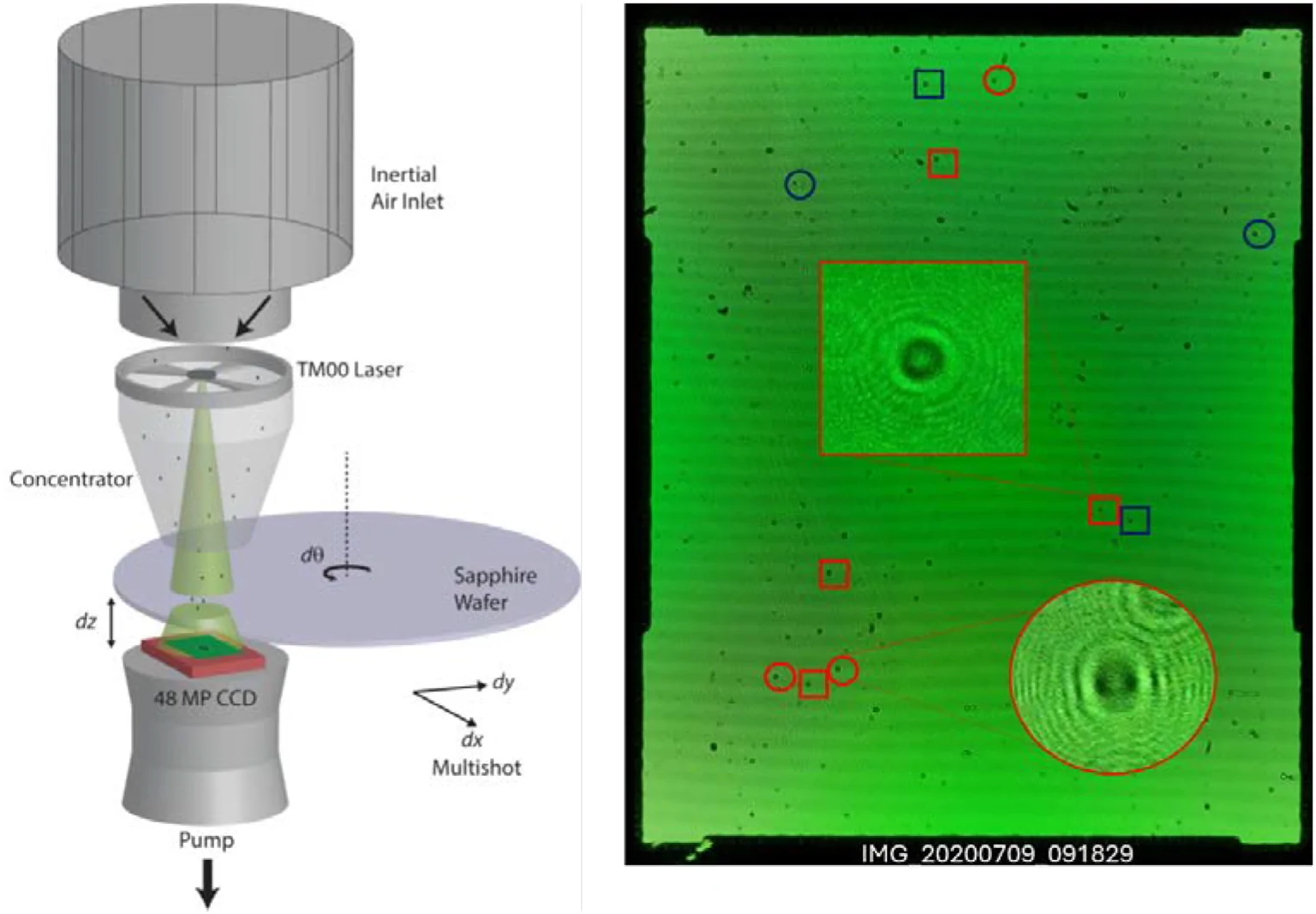
Schematic of digital holographic device (Wolf et al., 2020) and example of resulting holographic image with inserts of zoomed in holograms of both downy (square) and powdery (circle) mildew spores.

The sample is illuminated by a 510 nm single-mode diode laser, and the scattered light from the deposited particles interferes with the reference beam on a 48 MP camera sensor (Sony EXMOR IMX 586), creating a digital hologram (**Figure 1**). The laser has a nominal power of 10 mW and divergence angles of 8° and 22° (FWHM), parallel and perpendicular to the p-n junction, respectively. These characteristics translate to an intensity of ~ 0.2 mW/cm^2^ on the sensor. The 48 MP resolution of the CCD sensor allows the detection of up to hundreds of particles simultaneously, at high resolution (0.8 µm pixel pitch), as shown in the inserts of downy and powdery mildew spores in **Figure 1**. The analysis pipeline consists of (i) intensity normalisation to reduce illumination/background variation, (ii) automated detection of high-contrast diffraction patterns, (iii) extraction of square crops centered on each detected object, which are then resized to 220×220 pixels to standardise input, and (iv) classification using a two-stage CNN cascade described below (Section 2.2). Human-in-the-loop verification was used during early deployment to correct occasional misclassifications and to support iterative improvement of the labeled dataset.

With a distance between the sensor and the wafer of roughly 1 mm, the holographic pattern can be numerically inverted to reconstruct the 3D image of the object in the real space instead of the Fourier space using, *e.g.*, a variance optimisation or Fastnet for auto-focusing (Trujillo et al., 2020; Ren et al., 2018; Goodman, 2005). In the present case, however, the holograms are sufficiently distinctive to use the Fourier space images for AI recognition. Image acquisition, wireless data transfer, function control and energy management are carried out by an octa-core 2.6 GHz (max) processor and an ARM GPU card.

To relate segmented particle counts to approximate airborne concentrations, we performed a co-location campaign with a reference aerosol spectrometer (Grimm 1.109) to estimate an effective deposition/sticking factor under field conditions (**Supplementary Figure S1**). Based on this calibration, the current operational detection limit of the device is on the order of ~2 m^-3^ for a 3-hour acquisition window.

The core of the system is constructed in composite materials to avoid electric charge accumulation and water condensation. It is housed in a glass-fiber-loaded polymer enclosure and powered by solar panels. Images are uploaded via 4G cellular network to a centralised computer server for automated processing and near-real-time reporting. The instrument is fully stand-alone, can be placed anywhere in crop fields and is maintenance-free during a full season (**Figure 2**). A commercial version of this prototype will be available in a near future (https://arialight.ch).

**Figure 2:**
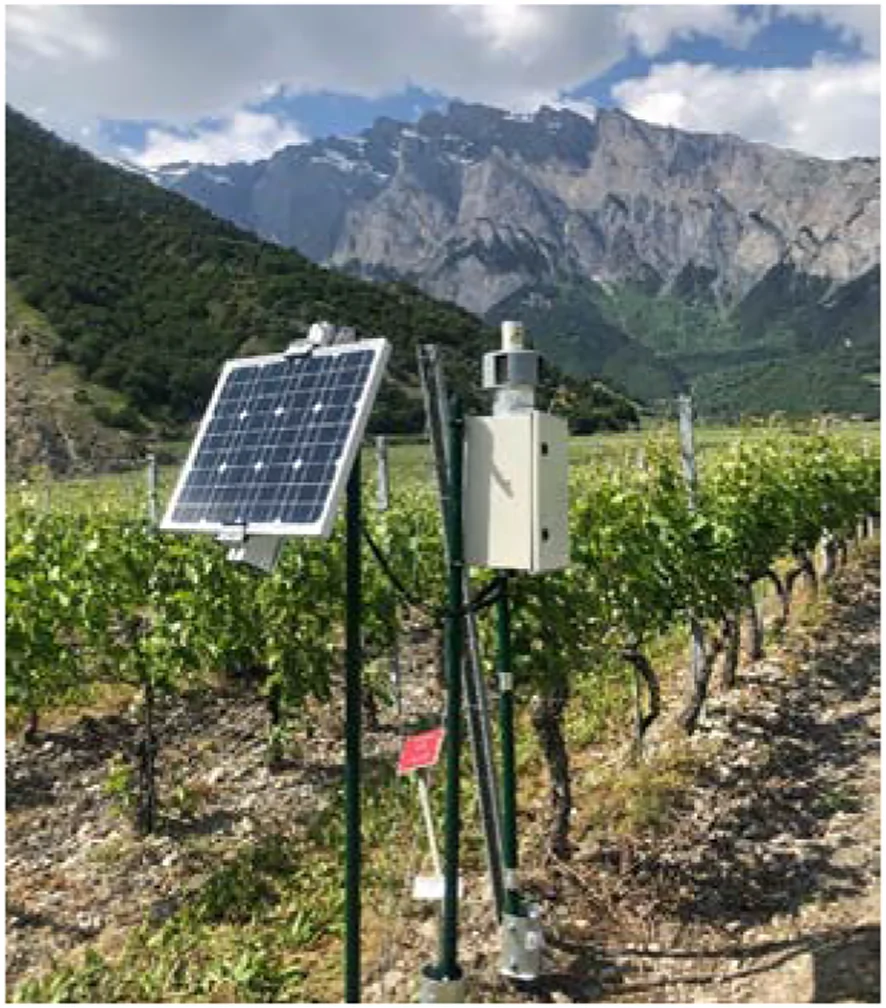
Picture of the stand-alone digital holographic device in the field in Switzerland.

### 2.2 Artificial-Intelligence-based identification of airborne fungal spores

The spore recognition relies on a synergistic approach involving two artificial intelligence algorithms. The AIs employ two-dimensional convolutional neural networks with three layers, trained over 1000 epochs using the ‘root mean square propagation’ (RMSProp) optimisation algorithm. Calculations are executed in MATLAB (Version R2023, The MathWorks, Inc., Massachusetts, USA), on an NVIDIA RTX A6000 GPU for efficient processing of both AI models. Architectures and training curves for these CNNs are provided in the **Supplementary Table S2** and **Supplementary Figure S3**. Our spore databases consist of 220 x 220-pixel images captured both in the laboratory and the field. For precise particle segmentation, we apply a binarisation method that extracts relevant features from the first diffraction ring while excluding those that deviate significantly in size or ellipticity from the holograms of the target spores. Additionally, we use artificial data augmentation techniques, including flips and translations, to expand the database. Before augmentation, the dataset collected from various samples over multiple acquisition sequences, described in the **Supplementary Tables S3**, was randomly split into 80% for training while the remaining 20% served for model verification. This ensured that there was no information leakage between the training and validation sets. To mitigate the potential influence of the database imbalance shown in **Supplemantary Tables S3**, class weighting was applied during the model training. The training and validation loss and accuracy curves in **Supplementary Figures S4** and **S5**, show convergence of the two models.

In addition to the two confusion matrices presented in **Figure 3**, the following detection metrics are used to evaluate the trained models, and the corresponding results are presented in **Supplementary Table S6**. Let TP denote the number of true positives, TN the number of true negatives, FP the number of false positives, and FN the number of false negatives. Precision, defined as TP/(TP + FP), represents the proportion of correct detections among all detections, while recall, defined as TP/(TP + FN), represents the proportion of correct detections among the samples that should be detected (Manning et al., 2008). The F1-score is the harmonic mean of precision and recall (Van Rijsbergen, 1979). Specificity, defined as TN/(TN + FP), represents the proportion of correct rejections among all negative samples (Green and Swets, 1966). The ROC-AUC corresponds to the area under the Receiver Operating Characteristic curve, which plots the TP rate against the FP rate, and reaches a maximum value of 1 for an ideal detector (Fawcett, 2006).

**Figure 3:**
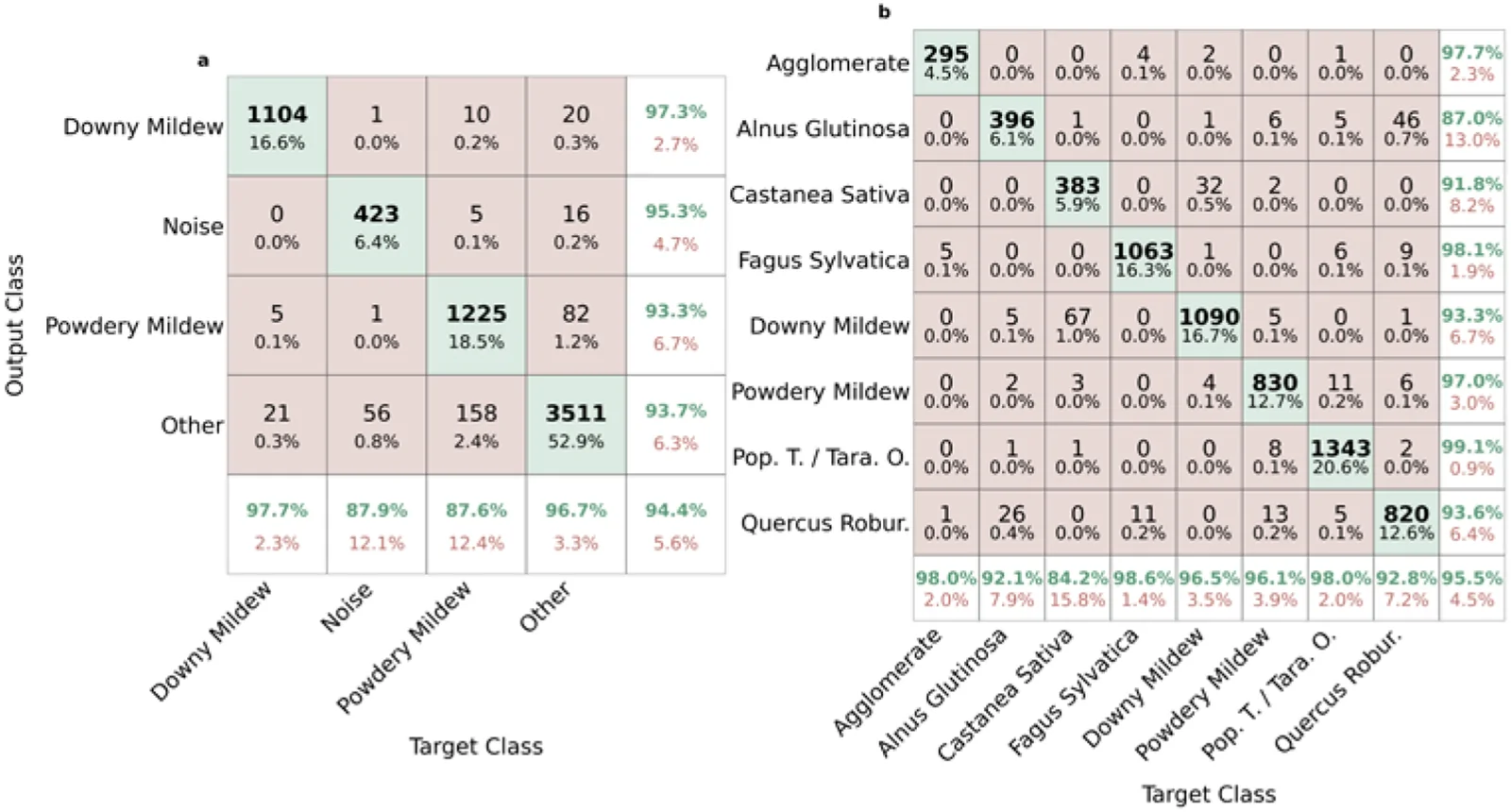
Confusion matrices of the 2 different AI used in our data treatment: **(a)** AI model trained on four classes with a mix of pure samples (*Downy* and *Powdery mildew* classes) and field (*Noise* and *Other* classes) images; **(b)** AI model with eight classes trained exclusively on pure laboratory samples.

The first AI model comprises four distinct classes (**Figure 3a**). The downy mildew and powdery mildew classes, which are the classes we aim to detect, exclusively consist of spores grown in controlled conditions and imaged with a laboratory setup. The second class, Noise, corresponds to particle aggregates, dirt traces, and other contaminants, equally serving as an indicator of particle collection wafer cleanliness. The class labeled Other represents various aerosols encountered in natural environments, sharing a similar size range to that of the spores of interest (10–30 mm). Data for these two classes predominantly originate from field campaign images.

The second AI model is more specific, encompassing eight classes (**Figure 3b**). Among these, five classes pertain to different pollen taxa: *Alnus glutinosa*, *Castanea sativa*, *Fagus sylvatica*, *Populus tremula/Taraxacum officinale*, and *Quercus robur*. These pollens were chosen because they were found to be interfering species with very similar patterns to either downy or powdery mildew and their holograms are visually indistinguishable. Additionally, they have a dissemination period corresponding to that of the two fungal pathogens (i.e. May to August). Specifically, it was found that *Castanea sativa* was a close interferent to downy mildew whereas *Quercus robur* resembled powdery mildew more closely, hence the distinction between these and the spores are particularly relevant in the confusion matrix. One of the five classes is a mix of two pollens; this is due to the strong similarity between the two species, the distinction of which is not relevant. Furthermore, a class representing pollen agglomerates, “clusters”, also observed in the field, is introduced. Finally, the remaining two classes focus on powdery and downy mildew spores, obtained from the same database as the first AI. Notably, this second AI is exclusively trained using laboratory-captured images. Pure samples have been grown specifically for this purpose at the Agroscope in Changins.

The two models operate sequentially, each serving a specific purpose: the first AI analyses each segment and identifies holograms that are likely to correspond to powdery or downy mildew spores. If a segment is identified as a spore, it proceeds to the second AI for further evaluation and classification. A particle is accepted as downy or powdery mildew only if both models independently assign the same class with a classification confidence score exceeding 75%. The first algorithm thus acts as a filter, with the ‘Other’ class trained on segments collected during previous measurement campaigns that are easily distinguishable from the two target spores. Despite the diverse range of particles within the “Other*”* class, its accuracy stands at 94.5% (**Figure 3a**). In contrast, the second AI is used to classify patterns that are challenging to differentiate visually and achieves a 95.5% accuracy in distinguishing these particles from one another (**Figure 3b**). Due to the diversity of atmospheric particles encountered under field conditions, the second AI cannot be used independently. However, combining it with the first AI allows us to specifically eliminate interfering particles of our two target pathogens.

### 2.3 Airborne spore collection and quantitative PCR

Airborne spores were collected using a SporeStick (OptiSense Ltd, UK) rotating-arm sampler installed in the vineyard canopy. The sampler was equipped with two wooden sticks coated with petroleum jelly and operated at 2400 rpm, corresponding to an estimated sampled air volume of 6.3 m3 per hour. Sticks were replaced three times per week, after 48 h to 72 h periods, transferred to sterile tubes, and stored at 8 °C until DNA extraction. DNA was extracted directly from the petroleum-jelly-coated sticks using a CTAB/phenol-chloroform protocol as described in (Douillet et al., 2022). Grapevine pathogen DNA was then quantified using a quadruplex quantitative PCR (qPCR) assay as described in (Leoni, 2026). Briefly, the qPCR targets *Plasmopara viticola*, *Erysiphe necator*, *Botrytis cinerea*, and an exogenous internal positive control. Reactions were performed in 20 µL per well using GoTaq Probe qPCR master mix (Promega, US), target-specific primers, and target-specific probes labeled with FAM, HEX, Cy5, and ROX. The qPCR program consisted of an initial hot-start step at 95 °C for 5 min, followed by 45 cycles of denaturation at 95 °C for 30 s and annealing/extension at 60 °C for 30 s, with fluorescence acquisition in the corresponding channels at each cycle after the annealing/extension step. Pathogen spore quantities were estimated from their specific standard curves as described in (Leoni, 2026).

## 3 Results

### 3.1 Comparison of holographic spore detection with multiplex qPCR

To independently validate the specificity and epidemiological relevance of the holographic detection under field conditions, the device was co-located with a volumetric sampling and multiplex quantitative PCR (qPCR) workflow in a Pinot Noir vineyard in Changins (Switzerland) during the 2024 season. qPCR sampling was performed at midday three times per week (Monday, Wednesday, Friday), while holographic measurements were recorded continuously and integrated over the corresponding time windows to enable direct comparison. qPCR assays targeted *Plasmopara viticola* and *Erysiphe necator* simultaneously (Leoni, 2026), and qPCR signals were converted to “estimated spore equivalents” using species-specific calibration curves derived from counted spores.

Absolute concentrations differed between the optical technique, which records and identifies spore morphology, and the molecular method which measures DNA sequences. In order to match the datasets, the holographic based data were summed up over the sampling periods of the rotorods used for the qPCR. In this way, we obtained 71 pairs of synchronous data corresponding to the same sampling time. The statistics were evaluated with R (on the log_10_ of the raw data), which resulted in Pearson: *r* = 0.68 *p* = <0.001 95%*CI* [0.534, 0.790] for *Plasmopara viticola* and Pearson: *r* = 0.45 *p* = <0.001 95%*CI* [0.245, 0.620] for *Erysiphe necator*, respectively. Similar differences were also previously reported in the literature (Apangu et al., 2023). The main reasons for the discrepancies are that (1) holography identifies structurally sound spores (as per viable and wet spores from pure cultures used in training) but may produce false positives from dry spores in outdoor conditions that have similar morphologies, (2) qPCR detects DNA from both viable spores and non-viable spore- or hyphal- fragments, and (3) the sampling methods are also different in our case (rotorod for the qPCR, semi-inertial deposition for the holographic device). Despite these differences, both methods captured similar temporal dynamics for downy and powdery mildew over the season, including periods of low background signal and pronounced epidemic peaks (**Figure 4**). This inter-comparison can thus be used primarily as a cross-validation of the temporal infection dynamics and the observation of risk-relevant threshold exceedance rather than direct quantitative statistical assessment.

**Figure 4:**
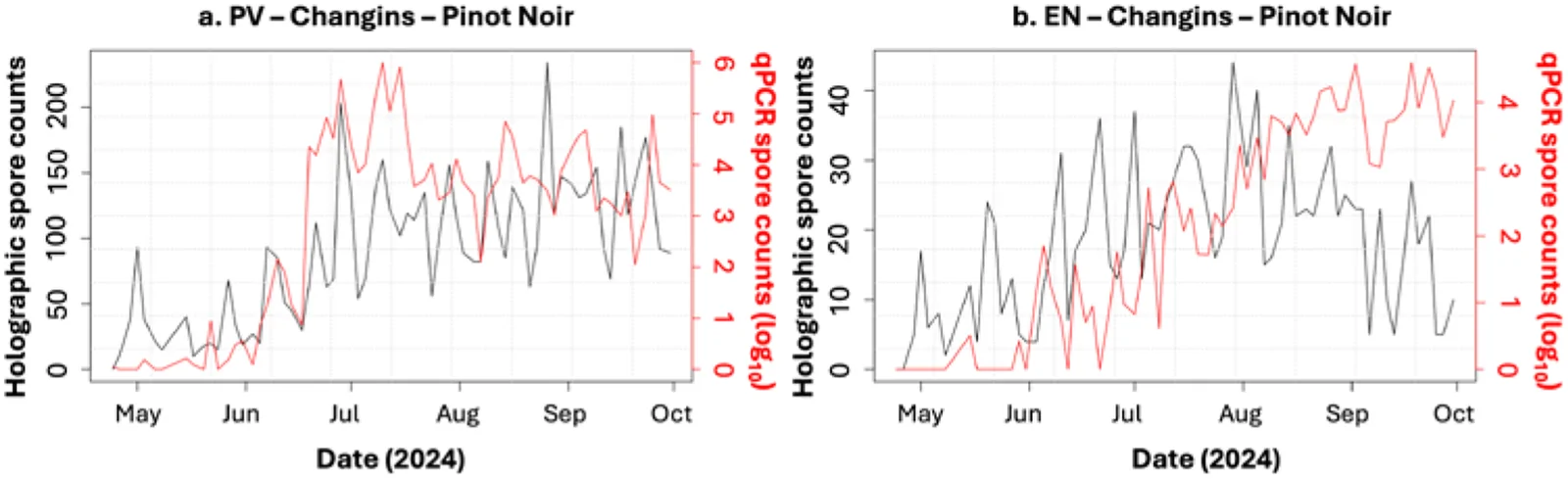
Holographic spore counts (black) and “estimated spore equivalents” qPCR values (red) are shown for **(a)** downy mildew (PV) and **(b)** powdery mildew (EN). QPCR sampling was performed at midday three times per week, and holographic data were integrated over the corresponding sampling windows. qPCR values represent estimated spore equivalents derived from specific-specific calibration curves.

### 3.2 Implementation of digital holography and AI for real-time pesticide management in Château Le Puy, Bordeaux, France in 2021 and 2022

The following results are presented as a case study conducted in the biodynamic vineyard of Château Le Puy, France (44°56’11.4”N 0°01’39.1”W) to evaluate how real-time airborne spore measurements can inform grower decision-making under operational conditions. The monitored parcel consisted of a fixed 0.7367-ha block comprising 34 rows of Merlot vines. The detector was installed within this parcel at approximately 1.5 m above ground. Downy mildew is typically the predominant disease affecting this parcel, whereas powdery mildew pressure is less pronounced; therefore, the data discussed in this section focus on downy mildew. Treatment frequency in the monitored parcel was compared with the standard management program applied to the remaining Merlot parcels of the estate, while comparative observations of disease status were made in the immediately adjacent Merlot parcel. The adjacent parcel was managed by the same winegrower under the same biodynamic production system and therefore provided the closest available operational reference. This comparison was not designed as a replicated controlled field experiment and is consequently interpreted as a case study of real-world implementation. Carrying out such a trial on a commercial vineyard requires certain compromises, such as the absence of an untreated control plot (which, in the majority of cases, is not desired by winegrowers, who may be wary of maintaining a source of production and spread of infectious spores throughout the season), and the need to facilitate the monitoring of diseases and the application of treatments without increasing the overall workload on the farm. Specifically, due to the distance, these observations could only be performed by personnel on site. Furthermore, the aim of these first two years of trials was to validate the operational viability of the real-time spore detector under real-world grape-production conditions without requiring an experimental setup that was overly sophisticated and time-consuming.

#### 3.2.1 Data access and treatment decisions

In the first year (2021), the device operated continuously to characterise airborne spore dynamics, while fungicide decisions remained primarily grower-led and weather-driven. For this purpose, data were recorded throughout the season but the treatments (based on copper, Cu, products against downy mildew) were not systematically applied according to the number of spores measured. In the second season (2022), data were provided in real time to allow the inclusion of pathogenic airborne spores in the treatment decisions. The treatment decisions followed a logic based on spore observations for a minimum of three consecutive days surpassing a threshold of 10 sporangia in 3 hours. The impact that the decisions had on the disease incidence was recorded by observing the symptoms.

#### 3.2.2 Treatments

The two seasons were very different, as seen in the precipitation and humidity measurements shown in **Figure 5a**. In 2021, the weather conditions were characterised by consistently high humidity levels, rarely dropping below 80% throughout the season. Additionally, numerous episodes of heavy rain were recorded, which were conducive to the development of downy mildew. Despite efforts to mitigate its impact through treatments, the infection persisted, resulting in a substantial presence of downy mildew spores in the air (**Figure 6a**). In contrast, in 2022, there were fewer and lighter rainfall events and consequently the relative humidity exhibited greater variability. These conditions resulted in lower disease pressure and provided wider treatment windows, allowing spore measurements to be more readily incorporated into treatment decisions. Thus, meteorological conditions and the implementation of spore-informed management both differed between the two seasons. The comparison between seasons should therefore be interpreted as an operational case study rather than as a controlled assessment of the independent effect of spore-informed management. **Table 1** shows the treatment schedule from June 2021 to mid-August. Early-season applications followed a conservative, phenology-driven protection strategy typical of commercial practice. The clearest opportunity for spore-informed treatment optimisation occurred later in the season, which is therefore the focus of **Table 1** and **Figure 6**.

**Figure 5:**
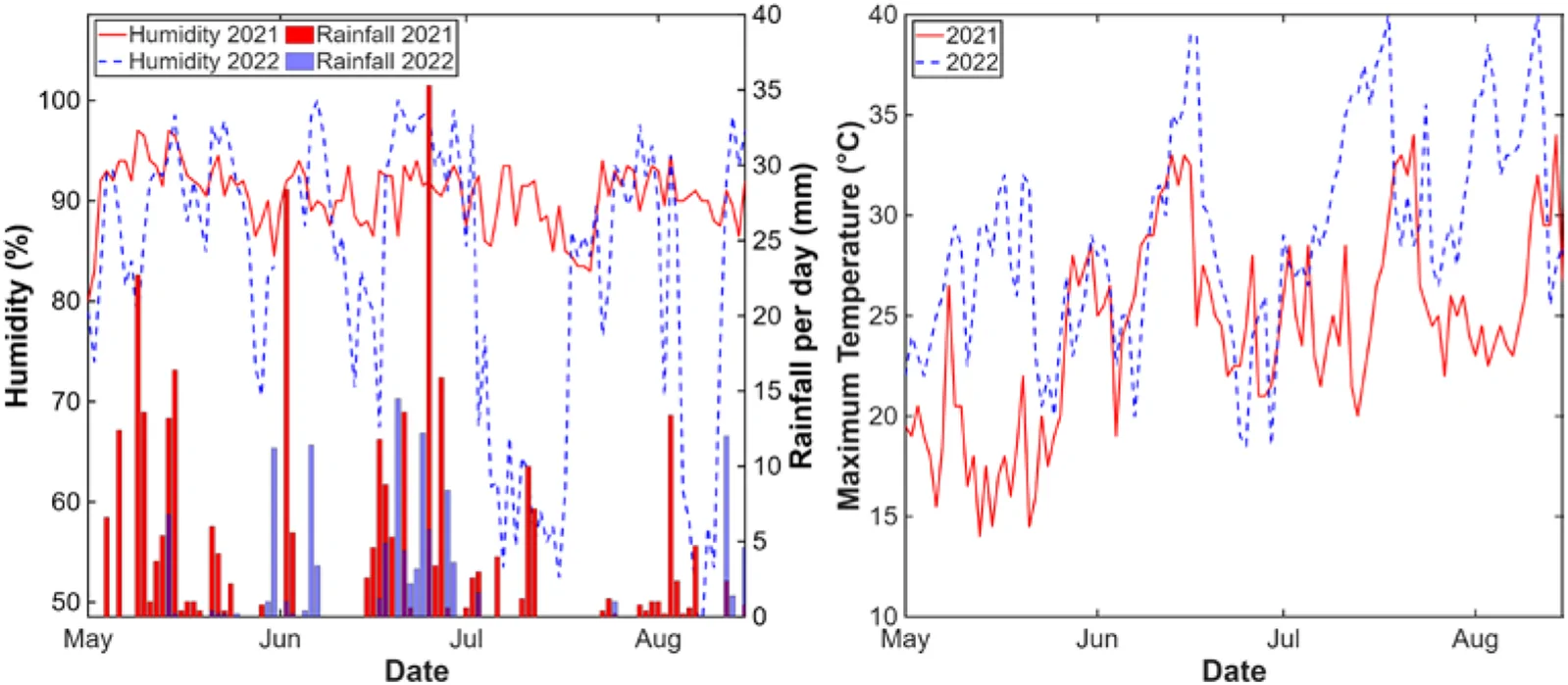
Weather data measured in 2021 and 2022 using a Météus Evolution weather station placed near the holographic detector at Château Le Puy. On the left is the daily rainfall and the relative humidity, and on the right, maximum daily temperatures. In 2021, the increased rainfall and persistent humidity corresponded to higher disease pressure compared to 2022 which was hotter and drier.

**Figure 6:**
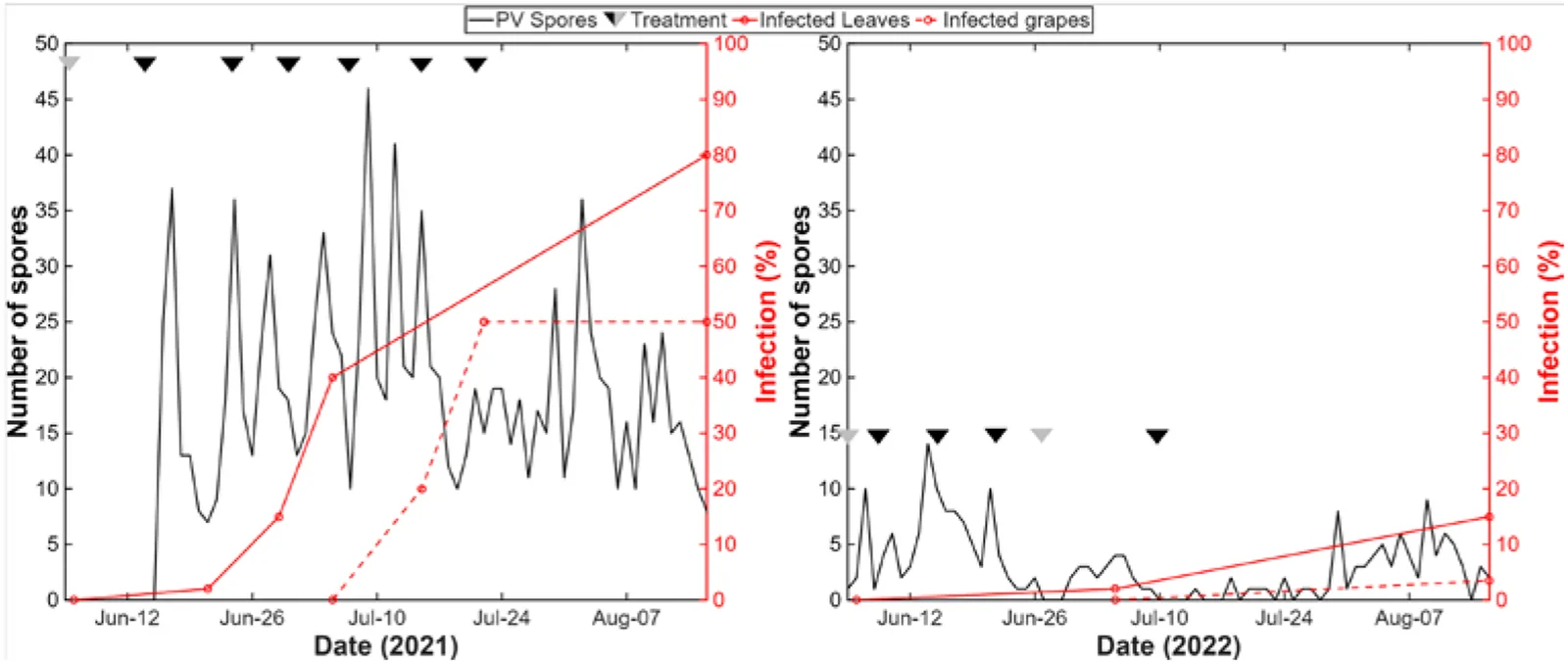
Graphs of maximum number of spores (black line) of PV measured at Château Le Puy on a given day for 2021 (left) and 2022 (right) as well as the observed infection percentage on both the leaves (solid red line) and grape bunch (dotted red line). Biodynamic treatment dates are included (black arrows) and grey arrows denote treatments applied elsewhere in the vineyard but not in the plot monitored with the holographic station.

**Table 1:** Treatment schedule and evaluations of the incidence of downy mildew on leaves and grape bunches (in percent) per plot; treatments in the test plot were decided based on a threshold of 10 detected sporangia in 3 hours, for three consecutive days.

2021			
Treatment date	Cu metal/Ha [gr]	Test Plot	Standard Program
07.06.2021	400		x
14.06.2021	400	x	x
24.06.2021	400	x	x
30.06.2021	400	x	x
07.07.2021	400	x	x
15.07.2021	400	x	x
21.07.2021	200	x	x
	Number of treatments	6	7
2022			
03.06.2022	200		x
08.06.2022	300	x	x
15.06.2022	300	x	x
22.06.2022	300	x	x
27.06.2022	200		x
10.07.2022	200	x	x
	Number of treatments	4	6

The rest of the domain’s merlot vines, the control, followed a standard treatment program based on the weather. Boxes have been ticked when treatment has been applied. May treatments are not displayed for clarity as they were the same for the entire domain.

#### 3.2.3 Treatment decision impact on downy mildew

Spore detections and the dates at which fungicides were applied for downy mildew in 2021 and 2022 are shown in **Figure 6**. Despite the challenging weather conditions for treatment scheduling in 2021, the monitored parcel received approximately 400 g/Ha less Cu metal than the rest of the domain, corresponding to a reduction of approximately 13%. However, the final yield in the test plot was comparable to the rest of the domain with a disease incidence of about 80% on leaves and 50% on grape bunches.

During the 2021 and 2022 campaigns, AI-generated detections were manually supervised, with approximately 15–25% of the reviewed detections requiring correction. This proportion varied over the season depending on environmental conditions and the presence of interfering particles. The corrected classifications were subsequently incorporated into the reference dataset to progressively improve the AI models. Despite this manual supervision, the AI-assisted workflow significantly accelerated the analysis, allowing updated data to be provided twice a day in 2022 and enabling timely information for treatment decisions.

Under this spore-informed management strategy, the monitored parcel received 30% less fungicide than the remaining Merlot parcels of the estate while maintaining comparable harvest yield and quality. The low disease incidence, where only 15% of leaves and 5% of grape bunches showed symptoms, further illustrates the interannual variation. As seen in **Figure 6**, following treatments, decreases in detected spore counts were generally observed over subsequent days, reflecting a combination of treatment effects and changing environmental conditions. For example, following treatment in late June 2022, spore concentrations remained low despite subsequent rainfall, and the monitored parcel did not receive the weather-model-recommended treatment of 27 June without an apparent subsequent increase in disease pressure. These observations illustrate how real-time spore measurements could support the omission of individual applications under the conditions encountered in 2022.

### 3.3 Systematic investigation of pesticide management at the agroscope test fields in Changins, Switzerland, 2023

A digital holographic spore detector was placed in a field in Changins, Switzerland (46°23’54.8”N, 6°13’52.5”E) at the edge of a row of Pinot Noir, at 1.50 m above ground, close to the top of the canopy. The year 2023 in Switzerland was characterised by very low downy mildew pressure; hence the data discussed in this section will focus on the powdery mildew epidemic. In contrast to the 2021–2022 campaigns, spore classification during the 2023 campaign was performed fully automatically, without manual supervision or correction.

At this stage of the project, the holographic detector enables the real-time capture of spores throughout the entire growing season. The next step is therefore to determine an approximate quantitative threshold for detected spores associated with a significant risk of fungal disease progression. The 2023 experimental set-up was therefore designed to (i) monitor the epidemiological dynamics of powdery mildew in the untreated control plot throughout the season, (ii) to make treatment decisions on individual plots, excluding the period of high susceptibility in the vines (flowering stage) during which standard practice recommends plant protection, and to assess the impact of each decision on disease progression, (iii) to carry out a retrospective study of spore capture dynamics in relation to individual treatment decisions.

#### 3.3.1 Disease assessments

During the growing season, disease assessments on leaves and grape bunches were conducted five times, ideally after a period allowing the consequences of each treatment decision to be assessed through the epidemiological evolution within each modality. The disease incidence is defined as the percentage of leaves or bunches showing powdery mildew symptoms of infection in relation to all organs observed. For each assessment, 3 x 100 leaves and 3 x 50 bunches per block were randomly scored. Incidence data were analyzed as proportions using a generalised linear model with binomial error distribution (infected vs. total), and multiple comparisons among blocks were performed using Tukey-adjusted contrasts (α = 0.05).

#### 3.3.2 Treatments

The experimental plot was divided into five distinct blocks (labeled with letters A-E), subject to different treatment decisions, based on the spore load measured by the holographic detector. The treatment schedule unfolded as follows: On 12th May, wettable sulfur (Thiovit Jet, Syngenta) was applied to blocks A and B. Subsequently, on 25th May, fluxapyroxad (Rondo Sky, Syngenta) was used on blocks B and E, followed by metrafenone (Vivando, BASF) on 7th June (all blocks except D). From the flowering stage until the end of the spraying season, uniform treatments were implemented across all blocks. Specifically, on 22nd June, fluxapyroxad was reapplied, and subsequent treatments occurred on 3rd, 14th, and 28th July, as well as on 11th August, using a combination of wettable sulfur and potassium hydrogen carbonate (Thiovit, Syngenta + Armicarb, Stähler). The comprehensive approach aimed to optimise disease control by targeting key applications with a significant impact on epidemiological dynamics within each treatment strategy. Early treatments corresponded to standard prediction-model-based decision points, enabling subsequent comparison between blocks receiving or omitting specific applications in relation to observed spore dynamics.

#### 3.3.3 Treatment decision impact on powdery mildew

The impact of contrasting fungicide sequences on powdery mildew incidence was evaluated in parallel with real-time airborne spore dynamics to identify which applications were epidemiologically decisive (**Figure 7** and **Table 2**).

**Figure 7:**
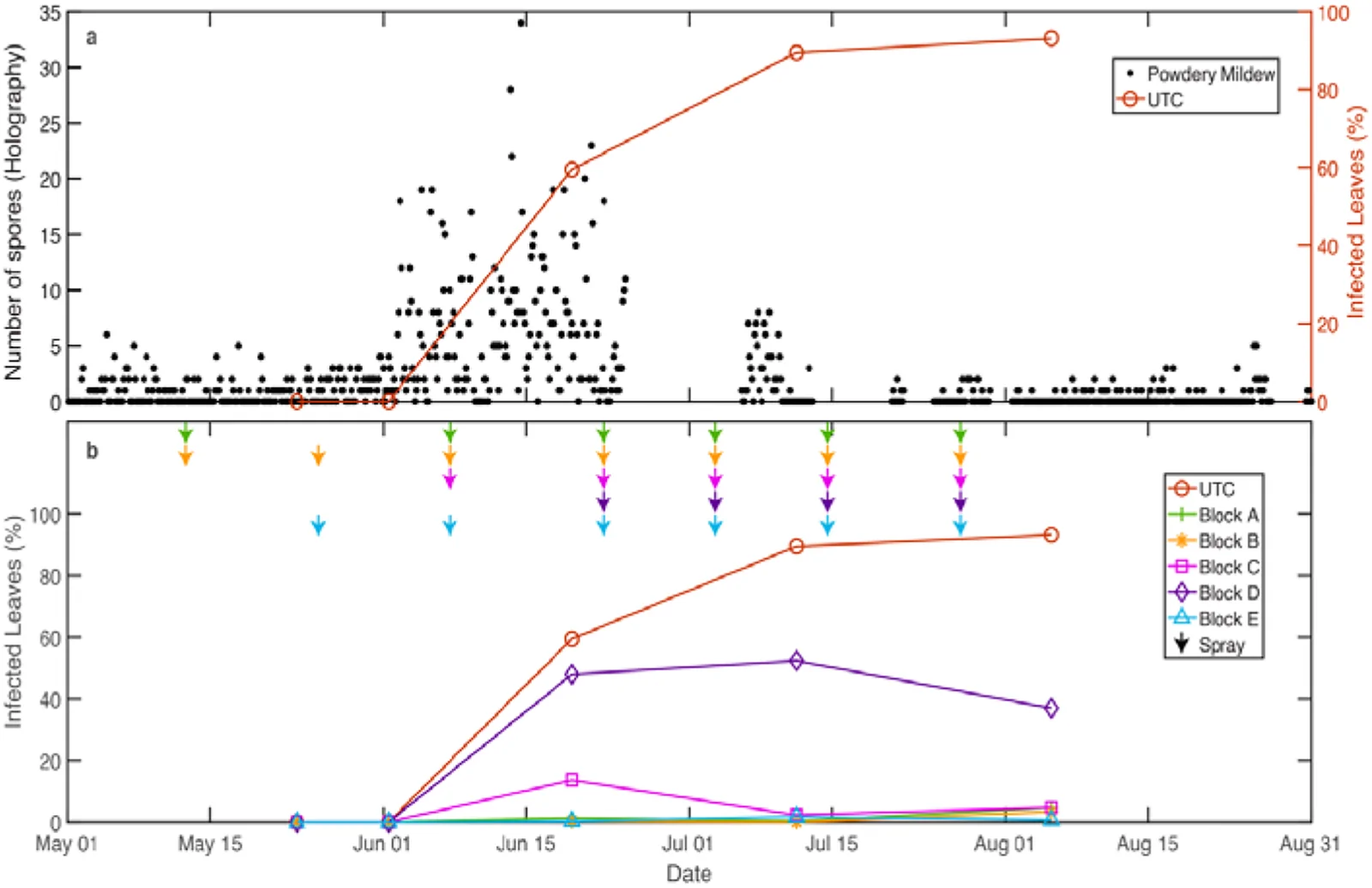
**(a)** Number of powdery mildew spores per 3 hours detected by the holographic detector (left vertical axis) and leaf infection intensity (right vertical axis) for the untreated control block (UTC) and **(b)** comparison between blocks receiving different fungicide spray treatments and schedules.

**Table 2:** Treatment schedule and evaluations of the incidence of powdery mildew on leaves (in percent) per block from A to E.

2023									
BBCH-scale	Treatment date	Product	Evaluation date	UTC	A	B	C	D	E
53	12.05.2024	Sulfur			x	x			
55			23.05.2024	0	0	0	0	0	0
55	25.05.2024	Fluxapyroxad				x			x
57			01.06.2024	0	0	0	0	0	0
59	07.06.2024	Metrafenone			x	x	x		x
69			19.06.2024	59.5^a^	1.3^c^	0.3^c^	13.7^bc^	48.0^ab^	0.3^c^
71	22.06.2024	Fluxapyroxad			x	x	x	x	x
73	03.07.2024	Sulfur + Bicar.			x	x	x	x	x
77			11.07.2024	89.5^a^	0.3^c^	0.0^c^	2.3^c^	52.4^b^	1.7^c^
77	14.07.2024	Sulfur + Bicar.			x	x	x	x	x
79	27.07.2024	Sulfur + Bicar.			x	x	x	x	x
83			05.08.2024	93.2^a^	4.7^c^	3.3^c^	4.8^c^	37^b^	0.7^c^
			**Number of treatments:**	**0**	**6**	**7**	**5**	**4**	**6**

Dedicated boxes have been ticked when treatment has been applied; UTC stands for the untreated control block. Statistical groups derived from ANOVA’s mean comparison of differences and Tukey’s test (Pr > 0.05) are represented by the lowercase letters a, ab, bc, and c together with the infection incidence of the block at BBCH-scale values 69, 77, and 83 (Rouder et al., 2016).

The exponential phase of powdery mildew appeared in the first fortnight of June and was evidenced by an approximately fivefold increase in particle counts on the holographic device. Each block represents a distinct treatment modality rather than a biological replicate. Given the layout of the plot, the *a priori* large number of decisions to treat based on epidemiological dynamics (apart from treatments at the flowering stage, which are carried out regardless of disease pressure as this is a period of high susceptibility for the vines), and in order to streamline the work, it was decided that the plot should be divided into large blocks representing a distinct treatment modality rather than a biological replicate. Consequently, the statistical analysis of the data will be subject to a bias that will be limited to a descriptive analysis of the impact of treatment decisions on the epidemiological dynamics. Differences among blocks therefore the epidemiological consequences of applying or omitting specific fungicide applications at defined time points, interpreted in conjunction with airborne spore dynamics. Despite the absence of spore data at the end of June, caused by a technical interruption, we can assume that environmental conditions were favorable for disease dynamics, as the untreated control reached 90% leaf incidence by mid-July. The rest of the season is marked by a significant reduction in disease pressure (number of conidia collected was close to background level), which explains the absence of any difference in efficacy between treatment strategies applied to bunches. However, the approach provides lessons in terms of identifying key treatments and, consequently, the number of treatments required over the season for optimal control of powdery mildew. The first two treatments occurred on May 12^th^ and May 25^th^, while the number of spores detected remained low (< 6 spores per image, every 3 hours), close to the background noise. Compared to the reference block B, blocks A and E, lacking one of the treatments, or block C, lacking the first two treatments, showed no statistically significant difference in their EN incidence during the intermediate and last evaluation at the end of the season on August 5^th^. The first peak of spores appeared on June 3^rd^ with 12 to 20 spores per image during this day. The treatment on 7 June showed that this application was crucial for the remainder of the season. Block D, which received its first treatment only on 22^nd^ June showed the highest disease incidence with 37% leaf incidence on August 5^th^. Spore quantities remained high throughout June, with a peak of 34 spores in 3 hours on June 16^th^.

Furthermore, the flowering period, considered to be the most critical for infection, was between June 12th and June 19th, and all blocks were treated afterwards. Despite a strong start of the epidemic, the hot and dry weather in July led to a decrease in disease pressure, which was associated with a decrease in spore counts until the end of the season. This field trial indicates that the fungicide application on June 7th, following the first marked spore peak, was critical for season-long disease control under the observed conditions, whereas earlier applications applied during periods of low airborne inoculum did not.

## 4 Discussion

### 4.1 Limitations of the study

The present study focuses on the development of a new methodology using laser digital holography and AI to identify and quantify pathogen spores in vineyards. It also includes two case studies, presented as examples, which do not constitute an established protocol applicable under all conditions. Further investigations in different fields, different seasons, different crops and different topographies are required in the future in order to assess the capabilities of this new methodology.

The AI-based recognition framework was intentionally developed for a defined diagnostic scope, the detection of *Plasmopara viticola* and *Erysiphe necator* within vineyards, where these pathogens dominate infection risk. Preliminary laboratory and field observations suggest that some non-target spores are morphologically distinct in holographic space, whereas others may require explicit inclusion in future training datasets. Expanding the reference library to additional taxa represents a natural extension of the system but does not detract from its current utility as a vineyard-scale decision-support tool. Improved false positive rejection relies on the inclusion of additional interferents in the reference database. To optimise the number of species, sampling and molecular analysis (or microscopy) could be carried out at the locations where holographic devices are implemented.

Continual learning may require adaptation to changes in climatic conditions, vineyard management practices, cultivars, sensor characteristics, or other environmental factors that may progressively affect model performance. Continued data collection over additional growing seasons and across different geographical regions will allow potential performance degradation to be identified. Periodic retraining or continual learning using newly collected and appropriately labeled data could then be considered to maintain model performance and adapt the models to changing conditions.

We also found that the positioning of the holographic device in the vineyards significantly influences the measured concentrations, and thus the adopted threshold values. For instance, simultaneous measurements of particles in the size range 10-20 µm using five optical particle sizers at different locations in a vineyard were carried out during a period of downy mildew pressure and revealed significant spatial heterogeneity over the field (Kilin et al., 2019). We plan to dedicate a specific campaign to this topic with a series of holographic devices in the future.

*Plasmopara viticola* is not currently known to be transported over very long distances by wind, and is thus considered relatively local in its dispersal (Gessler et al., 2011). The first outbreak may therefore occur more than 100 m from the holographic station and remain undetected. Depending on the topography and local weather conditions, there might be no likely outbreak point (*e.g.*, shaded and humid), where the station would have maximal probability to detect primary infection. In order to minimise this risk, the number of stations may be increased to cover the whole vineyard. In contrast *Erysiphe necator* does not require free water for survival which allows wind dispersion and more homogenous dispersal over long distances (Gadoury et al., 2012).

### 4.2 Potential of digital holography and AI in the context of smart agriculture

In the present study, we observed that airborne concentrations on the order of 5–10 sporangia/m³ are commonly associated with subsequent symptom development under favorable conditions for an epidemic development. In previous studies, similar threshold values were reported, ranging from 1 to 15 sporangia/m^3^, depending on the detection method (sampling and laboratory analysis via PCR, LAMP, microscopy), the grapevine cultivar, the location of the sampling device, and the weather conditions (Koledenkova et al., 2022; Brischetto et al., 2020; Hui Wang et al., 2023; Douillet et al., 2022).

In contrast to traditional sampling and laboratory analysis, the autonomous digital holographic device provides high-resolution, real-time spore detection, facilitating immediate and informed decision-making for disease management. The evolution of the concentration may provide a better proxy of the infection than a single-valued threshold, which is dependent on variables such as grapevine cultivar, device location and height, and foliage surface. The total number of detected spores for a 3-h sampling period ranges from 0 to 45 (0 to 90 sporangia/m^3^) for downy mildew and 0 to 35 (0 to 70 conidia/m^3^) for powdery mildew. A striking difference is observed for downy mildew between 2021 and 2022 in Bordeaux (the maximum was 28 sporangia/m^3^ in 2022), which can be attributed to very different weather conditions. Infection risk also depends on host phenology, microclimate, and recent treatment history, and therefore benefits from interpretation of spore dynamics over time rather than reliance on a single concentration value. High-frequency measurements enable identification of rising inoculum pressure and epidemic onset, which is particularly relevant for optimising treatment timing.

The AI-based recognition framework was intentionally developed for a defined diagnostic scope, the detection of *Plasmopara viticola* and *Erysiphe necator* within vineyard rows, where these pathogens dominate infection risk. Preliminary laboratory and field observations suggest that some non-target spores are morphologically distinct in holographic space, whereas others may require explicit inclusion in future training datasets. Expanding the reference library to additional taxa represents a natural extension of the system but does not detract from its current utility as a vineyard-scale decision-support tool.

The system is not intended to replace grower expertise or existing forecasting models, but to complement them by providing real-time biological information that is otherwise unavailable at the field scale. In the present study, treatment decisions ultimately remained under grower or experimental control, reflecting real-world deployment conditions where risk tolerance, logistics, and regulatory constraints influence management choices.

The potential economic relevance of the technology can be illustrated using a simplified prospective scenario. An order of magnitude for annual fungicide treatment costs, including labor, machinery and chemicals, is approximately €1000/ha (Testa et al., 2025). For a 10-ha vineyard, assuming a 30% reduction in treatment costs, this would correspond to savings of approximately €3000 annually.

The underlying holographic approach is not intrinsically restricted to grapevine pathogens. In principle, the system could be adapted to other airborne crop pathogens by acquiring representative reference samples and extending or retraining the classification models for the target organisms and relevant background aerosols. As an example of this broader applicability, data have also been collected over three growing seasons for apple scab (*Venturia inaequalis*), although these ongoing investigations are beyond the scope of the present study. Such adaptation would require pathogen- and crop-specific validation, particularly where particles have similar sizes and holographic signatures to abundant non-target biological particles. Different agricultural environments may also influence aerosol abundance, dispersal and sampling efficiency, while crop architecture and canopy structure may affect optimal device positioning. Finally, epidemiologically relevant spore concentrations and treatment-decision thresholds cannot be assumed to transfer directly between pathosystems or climatic regions and would need to be established through dedicated field studies.

## 5 Conclusion

By integrating real-time spore detection with established agronomic knowledge, digital holography-based monitoring may offer a practical pathway to optimize fungicide placement and potentially reduce fungicide inputs while maintaining effective disease control. The results presented here demonstrate that identifying key infection periods via airborne spore detection, rather than maintaining uniformly high treatment frequency, can substantially improve application efficiency. Beyond the vineyard pathosystems investigated here, the combination of autonomous bioaerosol monitoring, digital holography and AI could provide a broader framework for the surveillance of airborne crop pathogens. Continuous detection of changes in pathogen abundance could contribute to early disease warning systems by providing direct biological information before widespread symptoms become apparent. In the longer term, integration of such measurements with meteorological data, disease forecasting models and other field sensors could contribute to autonomous smart farming platforms in which crop-protection decisions are increasingly informed by real-time biological and environmental data. Continued validation across additional seasons, crops, pathogens and climatic regions will be required to establish pathogen-specific detection and decision thresholds and determine the broader applicability of this approach within precision agriculture.

## Data Availability

The datasets presented in this article are not readily available because they contain proprietary data related to technology currently under commercial development. The data necessary to verify the conclusions of the study may be made available by the corresponding author upon reasonable request, subject to applicable intellectual-property and confidentiality restrictions.

## Supplementary material

Supplementary Figure 1 Comparison of the total number of particles between 10-20 μm measured by the digital holographic device and the reference aerosol spectrometer Grimm 1.109 within the same plot of land in a vineyard line. This was used to estimate an effective deposition/sticking factor for concentration conversion.

Supplementary Table 2 Architecture of the K-classes CNNs (K is 4 or 8). Total number of parameters: 504+K.

Supplementary Table 3 Distribution of the data used for the 4-classes and 8-classes CNNs.

Supplementary Figure 4 Training and validation curves for the 4-classes CNN. X-axis are the steps, the batch size is 128, and the initial learning rate 5e-6.

Supplementary Figure 5 Training and validation curves for the 8-classes CNN. X-axis are the steps, the batch size is 256, and the initial learning rate 10e-6.

Supplementary Table 6 Metric results for the CNNs on the evaluation datasets.
