# Supplementary material for: Smart agriculture using digital holography and artificial intelligence
Source: Front Plant Sci. 2026 Sep 24;17:1871510. doi: 10.3389/fpls.2026.1871510 (PMC13646060; doi:10.3389/fpls.2026.1871510)
Supplement: Supplementary file 1 [file Supplementaryfile1.docx]

**Supplementary Information**

**Supplementary Figure S1.** Comparison of the total number of particles between 10-20 μm measured by the digital holographic device and the reference aerosol spectrometer Grimm 1.109 within the same plot of land in a vineyard line. This was used to estimate an effective deposition/sticking factor for concentration conversion.

| Name | Type | Filter Size | | Filters | Params |
| --- | --- | --- | --- | --- | --- |
| conv1 | nnet.cnn.layer.Convolution2DLayer | [3 3] | 8 | | 72 |
| BN1 | nnet.cnn.layer.BatchNormalizationLayer |  |  | | 0 |
| relu1 | nnet.cnn.layer.ReLULayer |  |  | | 0 |
| pool1 | nnet.cnn.layer.MaxPooling2DLayer |  |  | | 0 |
| conv2 | nnet.cnn.layer.Convolution2DLayer | [3 3] | 16 | | 144 |
| BN2 | nnet.cnn.layer.BatchNormalizationLayer |  |  | | 0 |
| relu2 | nnet.cnn.layer.ReLULayer |  |  | | 0 |
| pool2 | nnet.cnn.layer.MaxPooling2DLayer |  |  | | 0 |
| conv3 | nnet.cnn.layer.Convolution2DLayer | [3 3] | 32 | | 288 |
| BN3 | nnet.cnn.layer.BatchNormalizationLayer |  |  | | 0 |
| relu3 | nnet.cnn.layer.ReLULayer |  |  | | 0 |
| fc | nnet.cnn.layer.FullyConnectedLayer |  |  | | K |
| softmax | nnet.cnn.layer.SoftmaxLayer |  |  | | 0 |

**Supplementary Table S2.** Architecture of the K-classes CNNs (K is 4 or 8). Total number of parameters: 504+K.

| Label | Count | Percentage |
| --- | --- | --- |
| Mildiou | 5651 | 18.5 |
| Noise | 2405 | 7.9 |
| Oidium | 4319 | 14.1 |
| Other | 18145 | 59.5 |

| Label | Count | Percentage |
| --- | --- | --- |
| Agglomerate Fagus Sylvatica | 1506 | 4.5 |
| Alnus glutinosa | 2148 | 6.6 |
| Castanea Sativa | 2273 | 7.0 |
| Fagus Sylvatica | 5390 | 16.6 |
| Mildiou | 5651 | 17.4 |
| Oidium | 4319 | 13.3 |
| TaraxacumOfficinale PopulusTremula | 6853 | 21.0 |
| quercus robur | 4419 | 13.6 |

**Supplementary Tables S3.** Distribution of the data used for the 4-classes and 8-classes CNNs.

**Supplementary Figure S4.** Training and validation curves for the 4-classes CNN.
X-axis are the steps, the batch size is 128, and the initial learning rate 5e-6.

**Supplementary Figure S5.** Training and validation curves for the 8-classes CNN.
X-axis are the steps, the batch size is 256, and the initial learning rate 10e-6.

| Class | Precision | Recall | Specificity | F1-score | ROC-AUC |
| --- | --- | --- | --- | --- | --- |
| Mildiou | 0.9512 | 0.9841 | 0.9885 | 0.9674 | 0.9993 |
| Noise | 0.9303 | 0.8877 | 0.9943 | 0.9085 | 0.9966 |
| Oidium | 0.9906 | 0.9745 | 0.9985 | 0.9825 | 0.9994 |
| Other | 0.9788 | 0.9780 | 0.9689 | 0.9784 | 0.9966 |

| Class | Precision | Recall | Specificity | F1Score | ROC-AUC |
| --- | --- | --- | --- | --- | --- |
| Agglomerate Fagus Sylvatica | 0.9833 | 0.9801 | 0.9992 | 0.9817 | 0.9991 |
| Alnus glutinosa | 0.8683 | 0.9349 | 0.9900 | 0.9003 | 0.9959 |
| Castanea Sativa | 0.9177 | 0.8330 | 0.9944 | 0.8733 | 0.9934 |
| Fagus Sylvatica | 0.9842 | 0.9796 | 0.9969 | 0.9819 | 0.9994 |
| Mildiou | 0.9309 | 0.9664 | 0.9850 | 0.9483 | 0.9977 |
| Oidium | 0.9673 | 0.9583 | 0.9950 | 0.9628 | 0.9987 |
| TaraxacumOfficinale PopulusTremula | 0.9919 | 0.9832 | 0.9979 | 0.9875 | 0.9996 |
| quercus robur | 0.9326 | 0.9242 | 0.9895 | 0.9284 | 0.9940 |

**Supplementary Tables S6.** Metric results for the CNNs on the evaluation datasets.
